# Supplementary material for: Atypical pericapillary Ly6G⁺Nur77⁺ macrophages initiate type-2 immune responses to allergens in the mouse lung
Source: Nat Commun. 2026 Jan 22;17:1946. doi: 10.1038/s41467-026-68652-5 (PMC12929616; doi:10.1038/s41467-026-68652-5)
Supplement: Supplementary file 1 — Supplementary Information [file 41467_2026_68652_MOESM1_ESM.pdf]

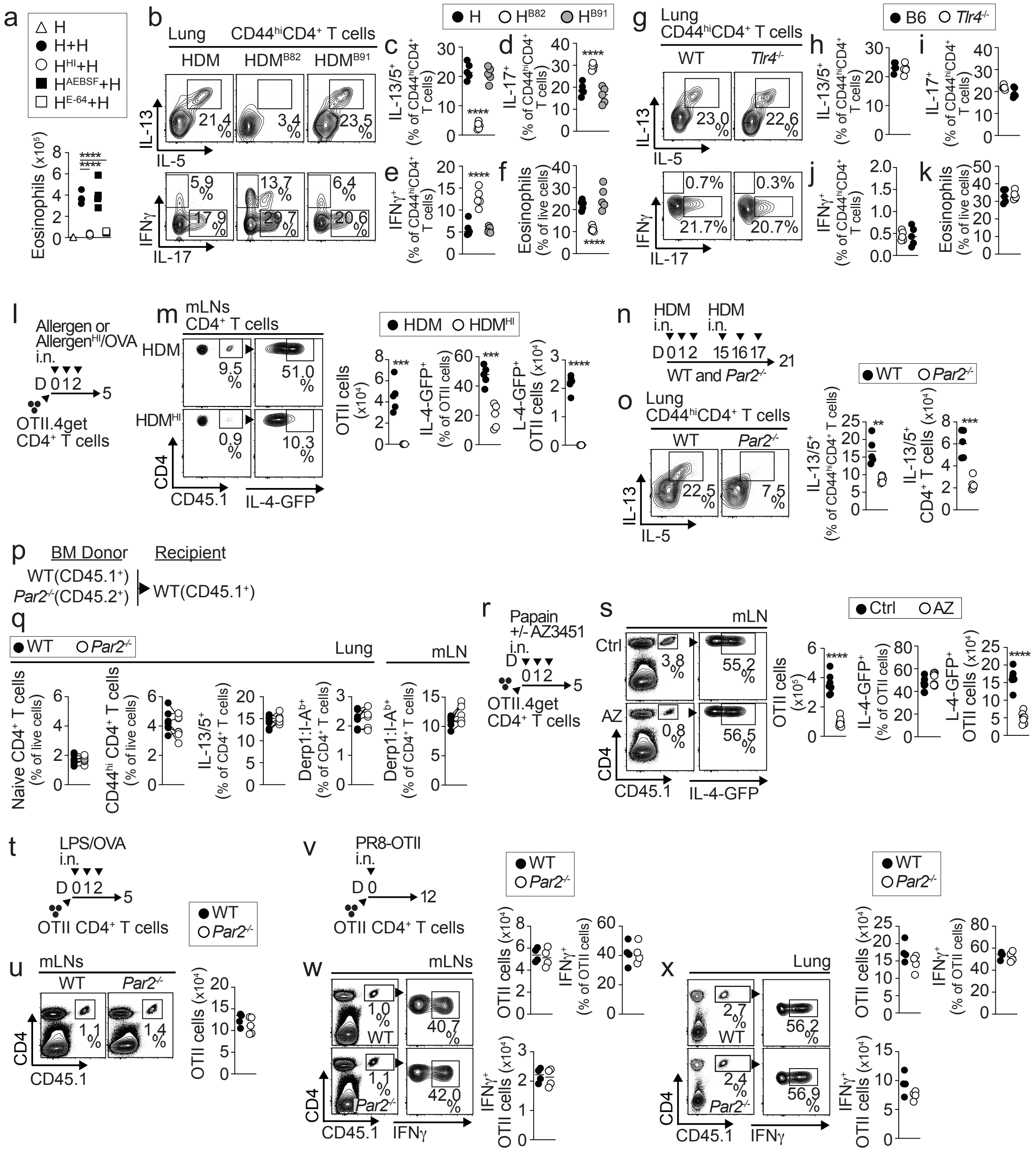

Supplementary Fig. 1: LPS and TLR4 do not contribute to type 2 responses to HDM, whereas PAR2 plays a crucial role in HDM immunity but does not influence responses to LPS or PR8 influenza.

a, Eosinophil quantification in the lungs of WT (B6) mice sensitized with native HDM (H), heat-inactivated HDM (H^{HI}), or HDM treated with protease inhibitors AEBSF (H^{AEBSF}) or E-64 (H^{E-64}), and challenged with HDM (sensitization n=3 mice, sensitization + challenge n=5 mice per group).

b-f, Representative flow cytometry plots (**b**) and quantification of IL-13/5$^+$ Th2 cells (**c**), IL-17$^+$ Th17 cells (**d**), IFNγ$^+$ Th1 cells (**e**), and eosinophils (**f**) in the lungs of WT (B6) mice sensitized and challenged with our regular low endotoxin HDM extract (<30 EU/mg endotoxin), as well as two commercially standardized HDM extracts: high endotoxin B82 (7,877 EU/mg endotoxin) and low endotoxin B91 (20 EU/mg endotoxin) (n=5 mice per group). **g-k**, Representative flow cytometry plots (**g**) and quantification of IL-13/5$^+$ Th2 cells (**h**), IL-17$^+$ Th17 cells (**i**), IFNγ$^+$ Th1 cells (**j**), and eosinophils (**k**) in the lungs of WT and *Tlr4*$^{-/-}$ mice sensitized and challenged with HDM (n=5 mice per group). **l,m**, Schematic of OTII cell transfer and allergen sensitization (**l**), and representative plots and quantification of donor OTII and IL-4-GFP$^+$ OTII cells from the mLN post-sensitization (**m**) (HDM n=5 mice, HDM^{HI} n=4 mice per group). **n,o**, Schematic of the HDM sensitization and challenge (**n**) and representative plots and quantification of Th2 cells in gated lung CD44^{hi}CD4$^+$ T cells (**o**) (n=5 mice per group). **p,q**, Schematic representation of BM chimeric mice (**p**) and quantification of the specified CD4$^+$ T cell populations within the CD45.1$^+$ (WT) and CD45.2$^+$ (*Par2*$^{-/-}$) compartments in the lung and mLN after HDM sensitization and challenge (**q**) (n=7 mice per group). **r,s**, Schematic of OTII cell transfer and papain sensitization in the absence or presence of PAR2 antagonist AZ3451 (**r**), and representative plots and quantification of donor OTII and IL-4-GFP$^+$ OTII cells from the mLN post-sensitization (**s**) (n=7 mice per group). **t,u**, Schematic representation of OTII cell transfer, LPS/OVA treatments, and analysis time point (**t**) and representative flow cytometry plots of donor OTII cells and corresponding quantification in the mLN (**u**) (n=5 mice per group). **v,x**, Schematic representation of OTII cell transfer, PR8-OTII infection, and analysis time point (**v**), representative flow cytometry plots of donor OTII cells and corresponding quantification of total OTII and IFNγ$^+$ OTII cells in the mLN (**w**) and lung (**x**) (n=4 mice per group). (**a,c,d,e,f**) Statistical tests are one-way ANOVA with Tukey's post hoc test. (**m,o,s**) Statistical tests are two-tailed unpaired t test. **p < 0.01; ***p < 0.001; ****p < 0.0001. Individual data shown in summary graphs represent biological replicates. Source data are provided in the Source Data file. Representative experiments of at least two performed.

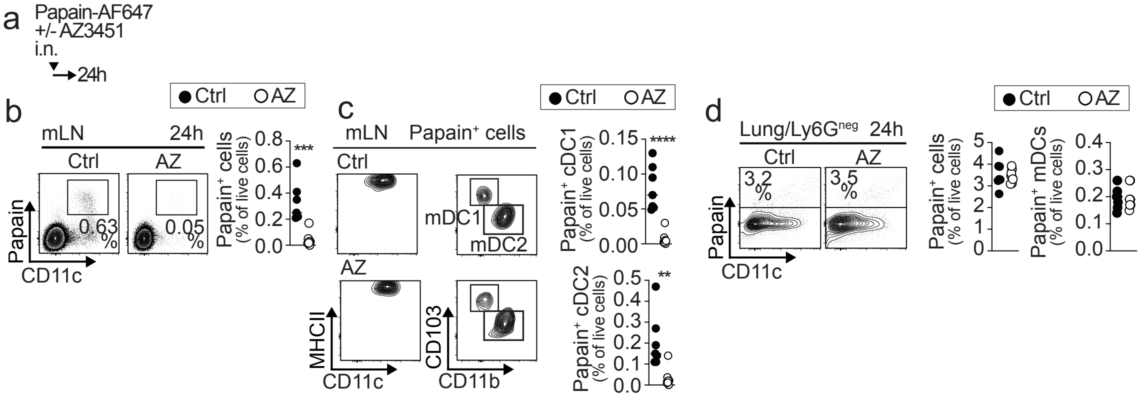

Supplementary Fig. 2: PAR2 signaling directs protease allergen–induced mDC migration to mLNs.

a-d, Schematic of papain sensitization with or without the PAR2 antagonist AZ3451 (**a**), and representative plots with quantification of papain$^+$ total cells, mDCs, mDC1, and mDC2 in mLN (**b,c**) and lung (**d**) (n=7 mice per group). Statistical tests are two-tailed unpaired t test. **p < 0.01; ***p < 0.001; ****p < 0.0001. Individual data shown in summary graphs represent biological replicates. Source data are provided in the Source Data file. Representative experiments of at least two performed.

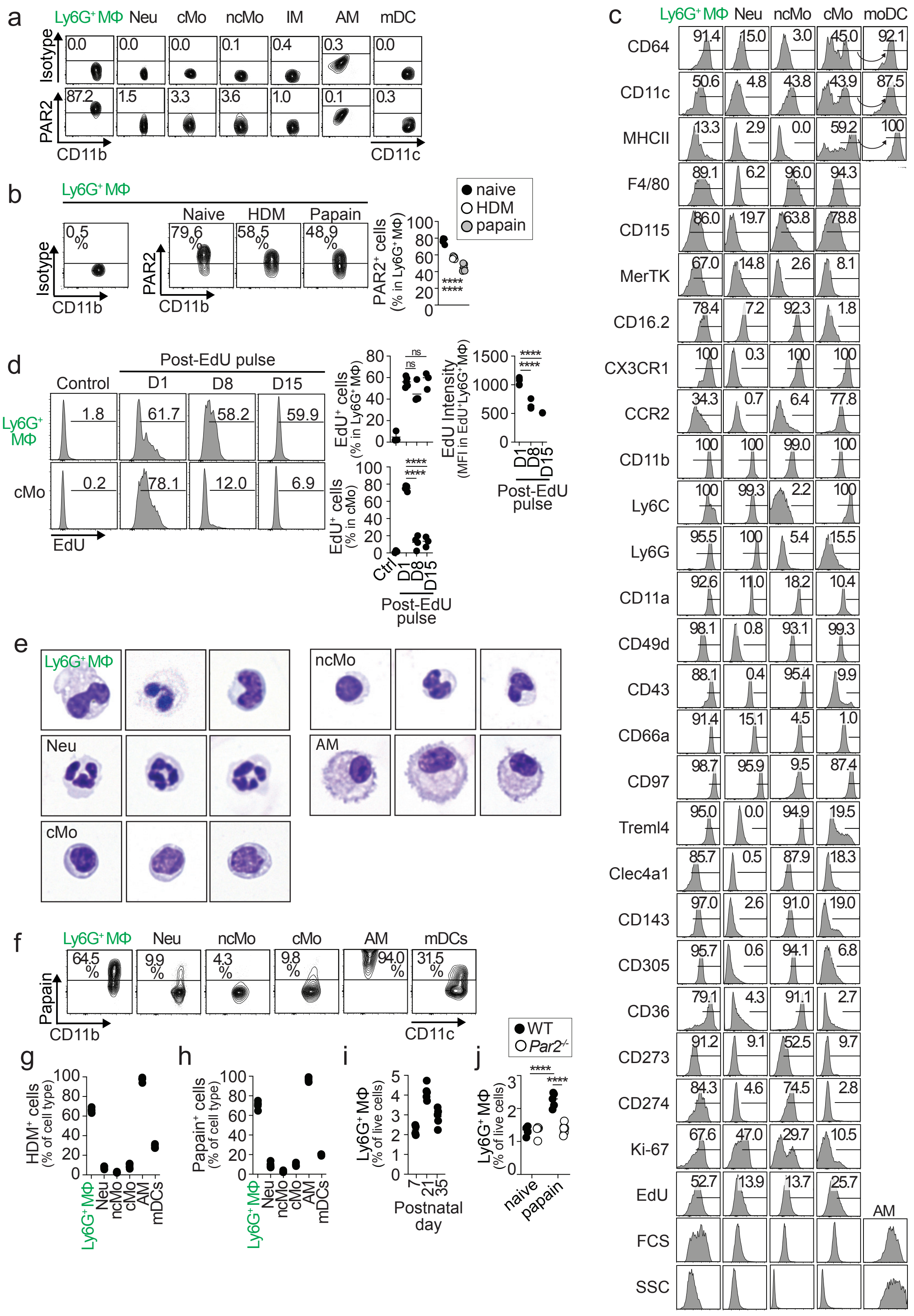
a
Ly6G+ MΦ Neu cMo ncMo IM AM mDC
Isotype
PAR2
CD11b
CD11c
0.0 0.0 0.0 0.1 0.4 0.3 0.0
87.2 1.5 3.3 3.6 1.0 0.1 0.3
b
Ly6G+ MΦ
Naive HDM Papain
Isotype
PAR2
CD11b
0.5 %
79.6 %
58.5 %
48.9 %
PAR2+ cells (% in Ly6G+ MΦ)
naive
HDM
papain
****
****
c
Ly6G+ MΦ Neu ncMo cMo moDC
CD64
CD11c
MHCII
F4/80
CD115
MerTK
CD16.2
CX3CR1
CCR2
CD11b
Ly6C
Ly6G
CD11a
CD49d
CD43
CD66a
CD97
Treml4
Clec4a1
CD143
CD305
CD36
CD273
CD274
Ki-67
EdU
FCS
SSC
AM
d
Control
Post-EdU pulse
D1 D8 D15
Ly6G+ MΦ
cMo
EdU
1.8 61.7 58.2 59.9
0.2 78.1 12.0 6.9
EdU+ cells (% in Ly6G+ MΦ)
ns
ns
EdU+ cells (% in cMo)
****
****
Ctrl D1 D8 D15
Post-EdU pulse
EdU Intensity (MFI in EdU+Ly6G+ MΦ)
****
****
D1 D8 D15
Post-EdU pulse
e
Ly6G+ MΦ
Neu
cMo
ncMo
AM
f
Ly6G+ MΦ Neu ncMo cMo AM mDCs
Papain
CD11b
CD11c
64.5 %
9.9 %
4.3 %
9.8 %
94.0 %
31.5 %
WT
Par2-/-
g
HDM+ cells (% of cell type)
Ly6G+ MΦ Neu ncMo cMo AM mDCs
h
Papain+ cells (% of cell type)
Ly6G+ MΦ Neu ncMo cMo AM mDCs
i
Ly6G+ MΦ (% of live cells)
7 21 35
Postnatal day
j
Ly6G+ MΦ (% of live cells)
****
****
naive papain

Supplementary Fig. 3: Ly6G$^+$ MΦ represents a phenotypically and morphologically distinct macrophage population in the lung that preferentially takes up allergens and expresses PAR2.

a,b, Representative flow cytometry plots and quantification of PAR2 expression (and isotype control) in the indicated lung cell populations from WT (B6) mice, either naïve or analyzed 24 h after allergen exposure (n=5 mice per group). **c**, Surface expression of the indicated markers, Ki-67 and EdU proliferation markers, and FSC/SSC profiles of the specified cell populations in the lung 24h after papain treatment. **d**, EdU pulse-chase experiment showing proliferative dynamics of Ly6G$^+$ MΦ compared with cMo. Mice were injected with EdU for two consecutive days, and lungs were analyzed at day 1, day 8, and day 15 after the last injection. Representative histograms of EdU fluorescence and quantification of the frequency of EdU$^+$ cells and mean fluorescence intensity (MFI) among EdU$^+$ cells in Ly6G$^+$ MΦ and cMo (Ctrl n=3 mice, day 1 n=5 mice, day 8 mice n=4 mice, day 15 n=3 mice per group). **e**, Wright-Giemsa staining of the indicated sorted lung cell populations. **f**, Representative flow cytometry plots of papain$^+$ cells within each indicated lung cell population 24h after exposure. **g,h**, Quantification of HDM$^+$ (**g**) and papain$^+$ (**h)** cells within each indicated lung cell population 24h after allergen exposure (n=4 mice per group). **i**, Kinetics of Ly6G$^+$ MΦ frequency in the lung at postnatal days 7, 21, and 35 (d7 n=4 mice, d21 and d35 n=5 mice per group). **j**. Quantification of Ly6G$^+$ macrophages in the lung before and after papain exposure in WT and Par2$^{-/-}$ mice (naïve n=4 mice, treated n=5-6 mice per group). Abbreviations: AM, alveolar macrophages; IM, interstitial MΦ; cMo, classical monocytes; ncMo, non-classical monocytes; Neu, neutrophils. Statistical tests are one-way ANOVA with Tukey's post hoc test. ****p < 0.0001. Individual data shown in summary graphs represent biological replicates. Source data are provided in the Source Data file. Representative experiments of at least two performed.

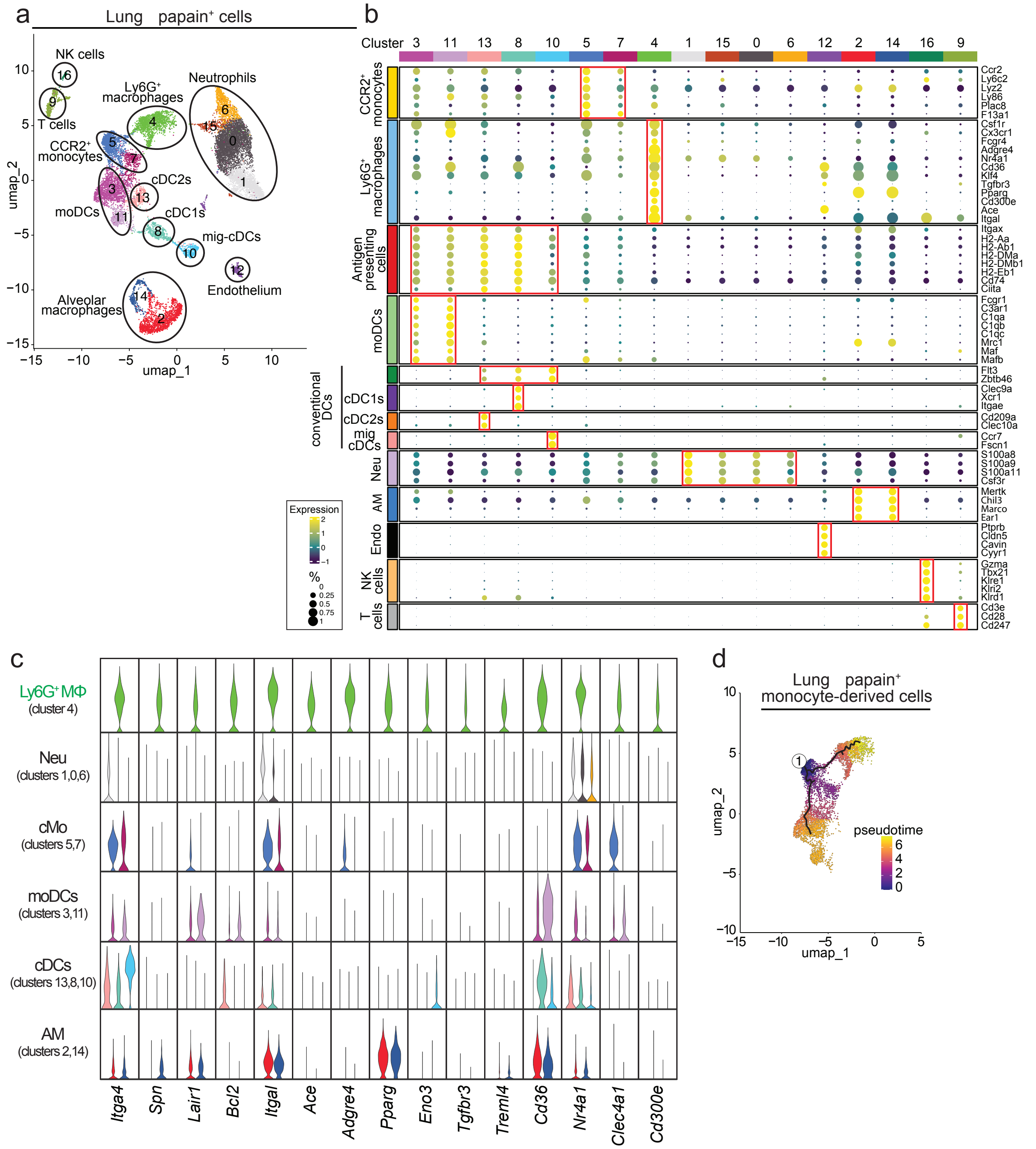

Supplementary Fig. 4: Ly6G⁺ MΦ represents a transcriptional distinct macrophage population in the lung.

a,b, Single-cell RNA-seq Uniform Manifold Approximation and Projection (UMAP) plot of lung papain⁺ cells sorted 24h after treatment (**a**), with annotated clusters based on signature genes shown in (**b**). **c**, Violin plots showing the expression of selected genes in the indicated clusters,

as shown in (**a**). **d**, Pseudotime analysis of scRNA-seq data from lung papain^{+} monocyte-derived cells, showing two independent trajectories originating from the CCR2^{+} classical monocyte cluster. dAbbreviations: AM, alveolar macrophages; cDCs, conventional DCs; cMo, classical monocytes; Endo, endothelium; Neu, neutrophils. Representative experiments of at least two performed.

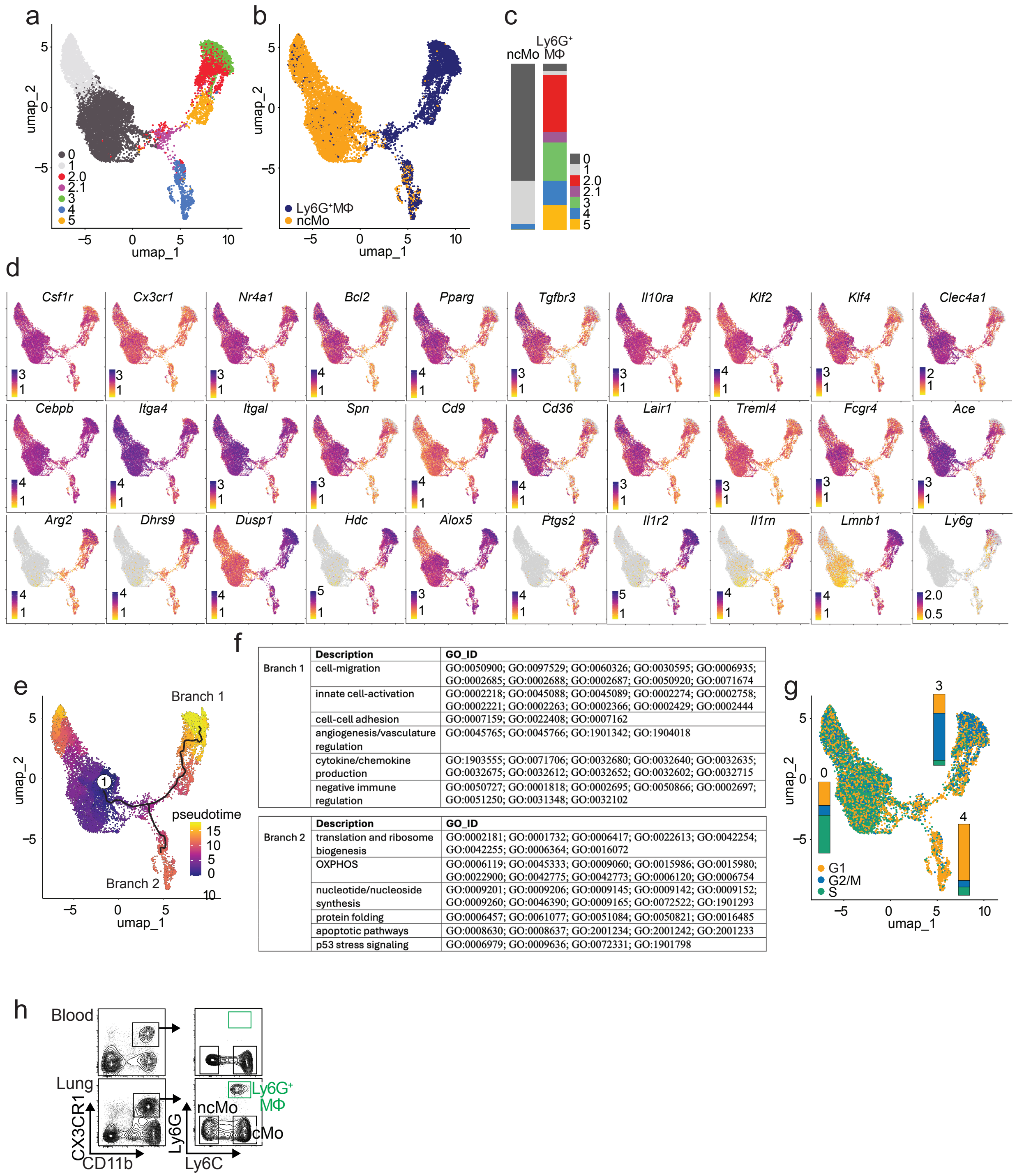

Branch 1	Description	GO_ID
	cell-migration	GO:0050900; GO:0097529; GO:0060326; GO:0030595; GO:0006935; GO:0002685; GO:0002688; GO:0002687; GO:0050920; GO:0071674
	innate cell-activation	GO:0002218; GO:0045088; GO:0045089; GO:0002274; GO:0002758; GO:0002221; GO:0002263; GO:0002366; GO:0002429; GO:0002444
	cell-cell adhesion	GO:0007159; GO:0022408; GO:0007162
	angiogenesis/vasculature regulation	GO:0045765; GO:0045766; GO:1901342; GO:1904018
	cytokine/chemokine production	GO:1903555; GO:0071706; GO:0032680; GO:0032640; GO:0032635; GO:0032675; GO:0032612; GO:0032652; GO:0032602; GO:0032715
	negative immune regulation	GO:0050727; GO:0001818; GO:0002695; GO:0050866; GO:0002697; GO:0051250; GO:0031348; GO:0032102

Branch 2	Description	GO_ID
	translation and ribosome biogenesis	GO:0002181; GO:0001732; GO:0006417; GO:0022613; GO:0042254; GO:0042255; GO:0006364; GO:0016072
	OXPHOS	GO:0006119; GO:0045333; GO:0009060; GO:0015986; GO:0015980; GO:0022900; GO:0042775; GO:0042773; GO:0006120; GO:0006754
	nucleotide/nucleoside synthesis	GO:0009201; GO:0009206; GO:0009145; GO:0009142; GO:0009152; GO:0009260; GO:0046390; GO:0009165; GO:0072522; GO:1901293
	protein folding	GO:0006457; GO:0061077; GO:0051084; GO:0050821; GO:0016485
	apoptotic pathways	GO:0008630; GO:0008637; GO:2001234; GO:2001242; GO:2001233
	p53 stress signaling	GO:0006979; GO:0009636; GO:0072331; GO:1901798

Supplementary Fig. 5: Integrated scRNA-seq and trajectory analysis reveal that Ly6G⁺ MΦ represent a distinct lineage developmentally related to non-classical monocytes.

a-c, Integrated UMAPs of isolated Ly6G⁺ MΦ and non-classical monocytes (ncMo) from the lung 24 h after papain exposure showing integrated clusters (**a**), distribution of Ly6G⁺ MΦ and ncMo

within the UMAP (**b**), and vertical bar plot depicting the proportion of clusters within each cell type (**c**). **d**, Featured UMAPs of shared and unique gene expression profiles. Ly6G$^+$ MΦ and ncMo shared a core gene set, including *Csf1r, Cx3cr1, Nr4a1, Bcl2, Pparg, Tgfbr3, Il10ra, Klf2, Klf4, Clec4a1, Cebpb, Itga4, Itgal, Spn, Cd9, Cd36, Lair1, Treml4, Fcgr4, and Ace.* Ly6G$^+$ MΦ uniquely expressed higher levels of *Arg2, Dhrs9, Dusp1, Hdc, Alox5, Ptgs2, Il1r2, Il1rn, Lmnb1, and Ly6g*. **e,f**, Pseudotime trajectory analysis using ncMo as the starting point reveals two branching differentiation pathways (**e**). Branch 1 is enriched for pathways associated with cell migration, activation, adhesion, angiogenesis, vasculature regulation, and cytokine/chemokine production. Branch 2 is enriched for pathways involved in translation, ribosome biogenesis, oxidative phosphorylation (OXPHOS), nucleoside/nucleotide synthesis, protein folding, apoptotic processes, and p53 stress signaling (**f**). **g**, Cell cycle phase analysis shows that cells differentiating along Branch 1 exhibit increased representation of G2/M-phase cells, consistent with active proliferation during Ly6G$^+$ MΦ development. **h**, Representative flow cytometry plots of blood and lung cells gated as indicated to identify Ly6G$^+$ MΦ, ncMo, and classical monocytes (cMo).

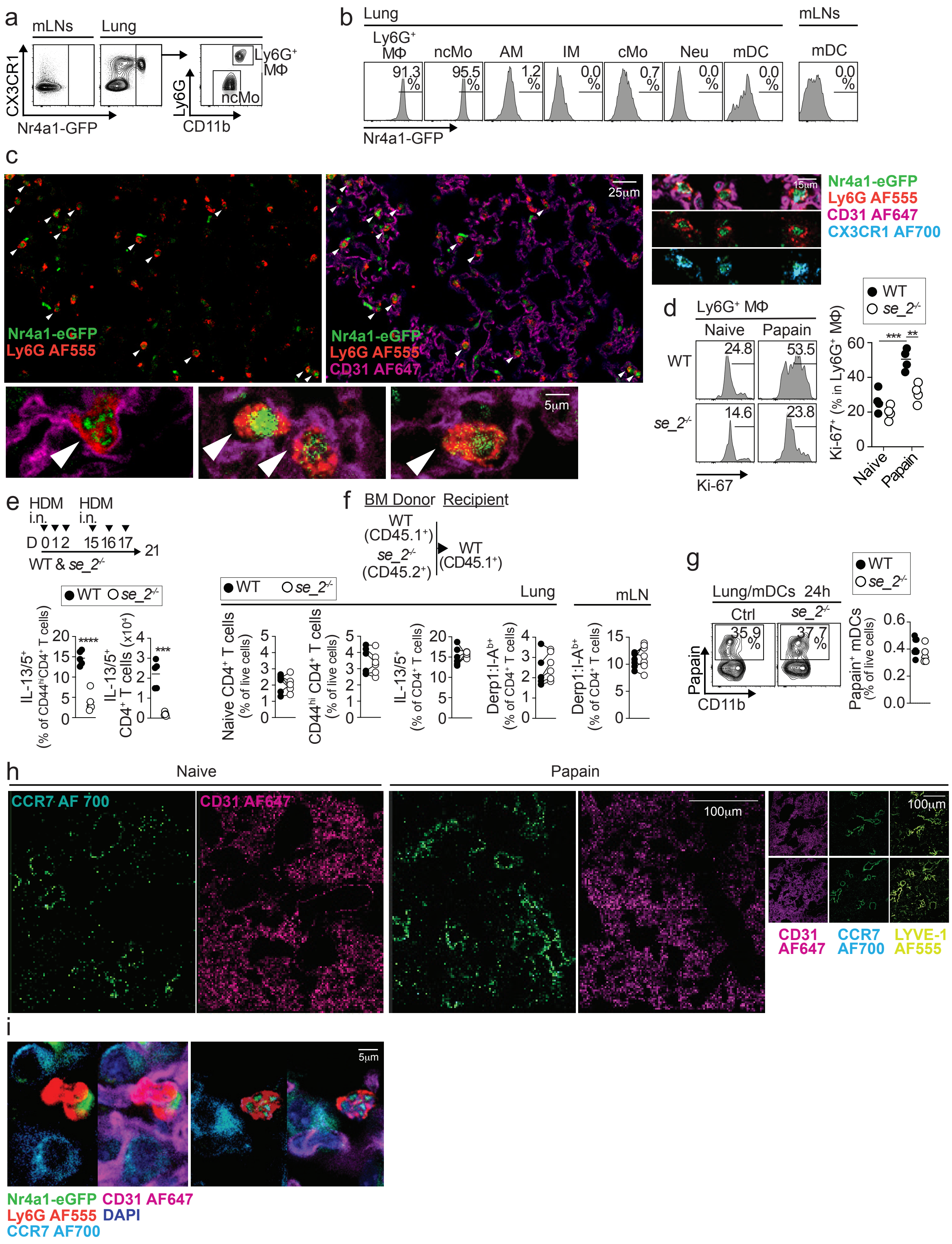

Supplementary Fig. 6: Ly6G⁺ MΦ uniquely express the transcription factor Nr4a1/Nur77, which is required for proliferation post-allergen exposure.

a, Representative flow cytometry plots of Nr4a1-GFP expression in mLNs and lung, showing gating and identification of $Nr4a1^+$ cells in Nr4a1/Nur77 reporter mice. **b**, Representative flow cytometry plots of Nr4a1-GFP expression in the indicated cell populations in the lungs and mLNs of Nr4a1/Nur77 reporter mice. **c**, Lung section of Nr4a1/Nur77 reporter mice stained to detect $Ly6G^+$ MΦ ($Nr4a1^+$, green; $Ly6G^+$, red; $CX3CR1^+$, light blue) and blood endothelial cells ($CD31^+$, purple). $Ly6G^+$ MΦ s are indicated by white arrows. **d**, Representative flow cytometry plots showing Ki-67 expression in $Ly6G^+$ MΦ from naïve and 24h papain-treated WT and *se_2*$^{-/-}$ mice (n=4 mice per group). **e**, Schematic of HDM exposure in WT and *se_2*$^{-/-}$ mice, and Th2 cell quantification in the lungs (n=5 mice per group). **f,** Schematic representation of BM chimeric mice and quantification of the specified $CD4^+$ T cell populations within the $CD45.1^+$ (WT) and $CD45.2^+$ (*se_2*$^{-/-}$) compartments in the lung and mLN after HDM sensitization and challenge (n=6 mice per group). **g**, Representative plots and quantification of $papain^+$ total cells and mDCs in the lungs of papain-exposed WT and *se_2*$^{-/-}$ mice (WT n=7 mice, *se_2*$^{-/-}$ n=5 mice per group). **h,i**, Lung sections of Nr4a1/Nur77 reporter mice stained to detect mDCs ($CCR7^+$, light blue), lymphatic endothelial cells (LYVE-1^+, yellow), blood endothelial cells ($CD31^+$, purple), and $Ly6G^+$ MΦ (*Nr4a1*$^+$, green; $Ly6G^+$, red) in naïve and papain-treated mice. Statistical tests are two-way ANOVA with Tukey's post hoc test (**d**) or two-tailed unpaired t test (**e**). **p < 0.01; ***p < 0.00; ****p < 0.0001. Abbreviations: AM, alveolar macrophages; IM, interstitial MΦ; cMo, classical monocytes; ncMo, non-classical monocytes; Neu, neutrophils. Individual data shown in summary graphs represent biological replicates. Source data are provided in the Source Data file. Representative experiments of at least two performed.

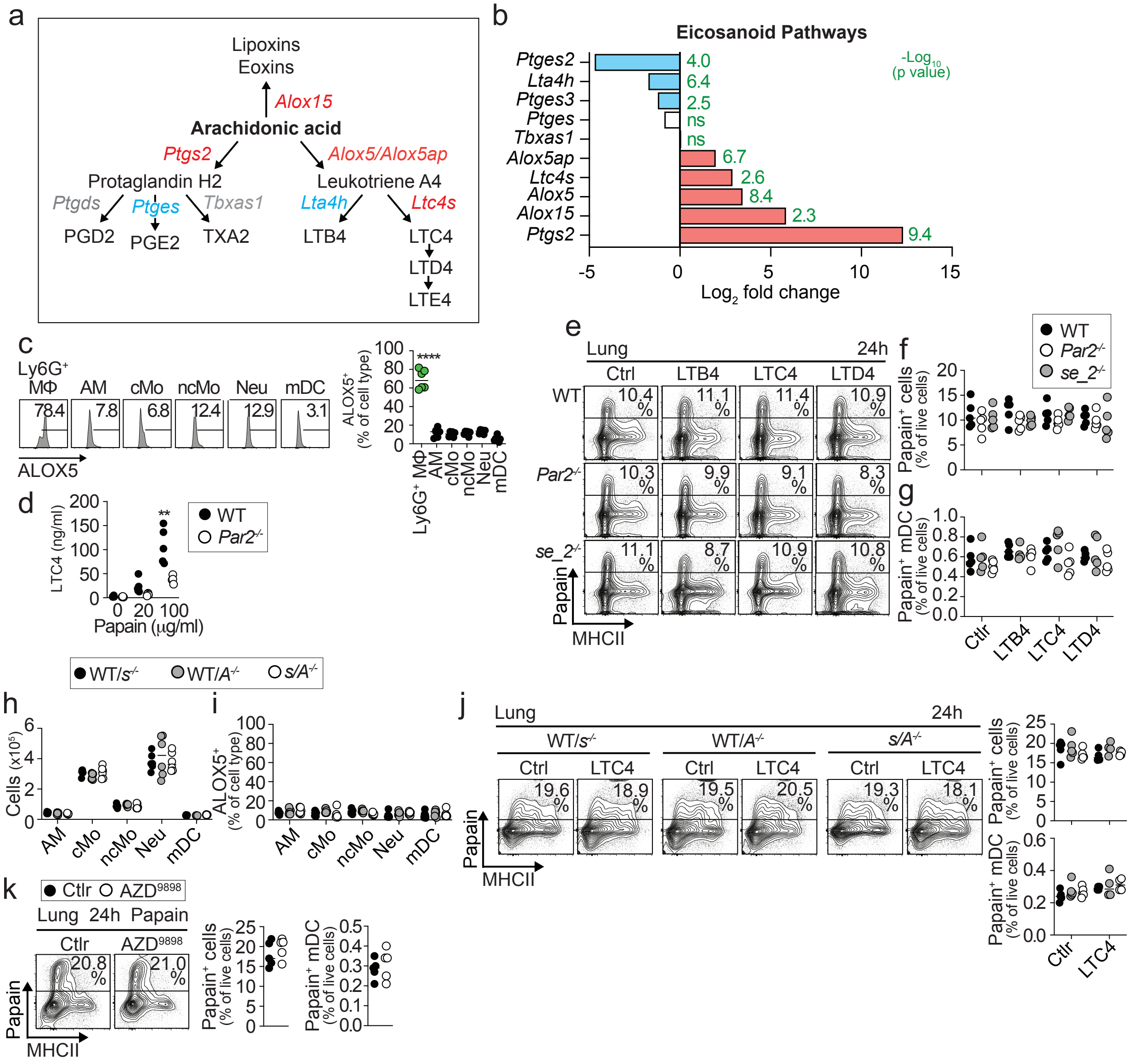

Supplementary Fig. 7: Ly6G⁺ MΦ express distinctive eicosanoid pathway genes.

a,b, Schematic representation of principal eicosanoid pathways from arachidonic acid (**a**). Genes in red indicate upregulation, genes in blue indicate downregulation, and genes in gray indicate no change in Ly6G⁺ MΦ compared to precursor CCR2⁺ bone marrow monocytes, as shown in (**b**) (pooled-mouse samples; n=4 per group). **c**, Representative flow cytometry plots depicting intracellular/intranuclear ALOX5 expression in the indicated lung cell populations from naïve WT (B6) mice (n=6 mice per group). **d**, LTC4 levels in culture supernatants from Ly6G⁺ MΦ isolated from WT and Par2⁻/⁻ mice and stimulated *in vitro* with papain (pooled-mouse samples; n=3-5 per group). **e-g**, Representative plots (**e**) and quantification of papain⁺ total cells (**f**) and mDCs (**g**) in

the lungs of WT, *Par2*$^{-/-}$, and *se_2*$^{-/-}$ mice, 24h after allergen treatment, with and without co-treatment with LTB4, LTC4, or LTD4 (n=5 mice per group). **h-j**, Cell quantification (**h**) and intracellular/intranuclear ALOX5 expression (**i**) (n=6 mice per group) in the indicated lung cells 24h after papain exposure in WT/*s*$^{-/-}$, WT/*A*$^{-/-}$, and *s*/*A*$^{-/-}$ chimeric mice, and representative plots and quantification of papain^{+} total cells and mDCs in the lungs of WT/*s*$^{-/-}$, WT/*A*$^{-/-}$, and *s*/*A*$^{-/-}$ chimeric mice, 24h after allergen treatment, with and without co-treatment with LTC4 (**j**) (Ctrl n=5, LTC4 n=4 mice per group). **k,** Representative plots and quantification of papain^{+} total cells and mDCs in the lungs of papain-exposed mice co-treated or not with the LTC4S inhibitor AZD9898 (n=5 mice per group). (**c**) Statistical tests are one-way ANOVA with Tukey's post hoc test. (**d**) Statistical tests are two-tailed unpaired t test. $^{**}p < 0.01$; $^{****}p < 0.0001$. Abbreviations: AM, alveolar macrophages; cMo, classical monocytes; ncMo, non-classical monocytes; Neu, neutrophils. Individual data shown in summary graphs represent biological replicates. Source data are provided in the Source Data file. Representative experiments of at least three performed.

Supplementary Table 1. Antibodies used for flow cytometry and immunostaining

Target	Clone	Supplier	Catalog #	RRID	Application	Dilution
ALOX5	ARC1926	Invitrogen	MA5-38050	AB_2897968	Intracellular	1:100
B220	RA3-6B2	BD Biosciences	553093	AB_394622	Surface	1:200
CCR2	475301	R&D Systems	FAB5538A100	—	Surface	1:200
CCR7	4B12	eBioscience	13-1971-82	AB_466642	Surface	1:200
CD103	M290	BD Biosciences	557495	AB_396732	Surface	1:200
CD11a	M17/4	BD Biosciences	741071	AB_2870679	Surface	1:800
CD11b	M1/70	BD Biosciences	553311 / 562127	AB_396680 / AB_10896991	Surface	1:200
CD11c	HL3	BD Biosciences	553800 / 558079 / 563048	AB_395059 / AB_647251 / AB_2734778	Surface	1:200
CD115	AFS98	eBioscience	13-1152-85	AB_466564	Surface	1:200
CD143	230214	R&D Systems	FAB15131R	—	Surface	1:200
CD16.2 (FcγRIV)	—	BioLegend	149512	AB_2632745	Surface	1:400
CD273 (PD-L2)	TY25	BioLegend	107218	AB_2728126	Surface	1:200
CD274 (PD-L1)	10F.9G2	BioLegend	124311	AB_10612935	Surface	1:200
CD305 (LAIR-1)	113	eBioscience	12-3051-82	AB_1210738	Surface	1:200
CD31	MEC 13.3	BioLegend	102516	AB_2161029	Surface	1:200
CD36	CRF D-2712	BD Biosciences	562702	AB_2737732	Surface	1:200
CD4	GK1.5	BD Biosciences	553729	AB_395013	Surface	1:200
CD4	RM4-5	BD Biosciences	550954 / 563726	AB_393977 / AB_2738389	Surface	1:200
CD43	S7	BD Biosciences	747726	AB_2872201	Surface	1:400
CD44	IM7	BD Biosciences	560780	AB_1937328	Surface	1:200
CD45.1	A20	BD Biosciences	553775 / 558701	AB_10926208 / AB_1645214	Surface	1:200
CD45.2	104	BD Biosciences	560696 / 558702 / 563685	AB_1727494 / AB_1645215 / AB_2738374	Surface	1:200
CD49d	R1-2	BioLegend	103621	AB_2565776	Surface	1:200

Target	Clone	Supplier	Catalog #	RRID	Application	Dilution
CD64	X54-5/7.1	BioLegend	139311 / 139306	AB_2563846 / AB_11219391	Surface	1:100
CD66a	CC1	BD Biosciences	750880	AB_2874976	Surface	1:200
CD88	20/70	BioLegend	135810	AB_10900812	Surface	1:200
CD97	587702	BD Biosciences	747935	AB_2872396	Surface	1:200
CX3CR1	SA011F11	BioLegend	149005	AB_2564314	Surface	1:200
DAPI	—	Fisher Scientific	BDB564907	—	Nuclear stain	1:1000
DCIR4	MH7E7	BD Biosciences	751754	AB_2875731	Surface	1:200
F4/80	BM8	eBioscience	11-4801-82 / 12-4801-82	AB_2637191 / AB_465923	Surface	1:100
GFP	—	Life Technologies	A21311	AB_221477	Intracellular	1:500
I-A/I-E	M5/114.15.2	BioLegend	107620	AB_493527	Surface	1:1500
IFN-γ	XMG1.2	BD Biosciences	557649	AB_396766	Intracellular	1:100
IL-13	eBio13A	eBioscience	12-7133-82	AB_763559	Intracellular	1:100
IL-17	TC11-18H10.1	BioLegend	506941	AB_2565836	Intracellular	1:100
IL-5	TRFK5	BioLegend	504311	AB_2563161	Intracellular	1:100
Ki-67	16A8	BioLegend	652406	AB_2561930	Intracellular	1:100
Ly6C	AL-21	BD Biosciences	560596	AB_1727555	Surface	1:400
Ly6G	1A8	BD Biosciences / BioLegend	560601 / 127603	AB_1727562 / AB_1186105	Surface	1:400
MertK	DS5MMER	eBioscience	17-5751-82	AB_2716943	Surface	1:100
PAR2	SAM11	Santa Cruz Biotechnology	sc-13504	AB_628101	Surface	1:100
Siglec-F	E50-2440	BD Biosciences	565526 / 565183	AB_2739281 / AB_2739097	Surface	1:200
Siglec-H	440c	BD Biosciences	74767	AB_2744232	Surface	1:200
Streptavidin	—	Fisher Scientific	565144 / 560797 / S-32355	AB_2869657 / AB_2033992	Secondary	1:200
Treml4	16E5	BD Biosciences	569880	AB_3685339	Surface	1:200
