# Supplementary material for: Atypical pericapillary Ly6G⁺Nur77⁺ macrophages initiate type-2 immune responses to allergens in the mouse lung
Source: Nat Commun. 2026 Jan 22;17:1946. doi: 10.1038/s41467-026-68652-5 (PMC12929616; doi:10.1038/s41467-026-68652-5)
Supplement: Supplementary file 2 — Descriptions of Additional Supplementary Files [file 41467_2026_68652_MOESM2_ESM.pdf]

Descriptions of Additional Supplementary Files

Supplementary Data 1. Signatures of monocyte subsets used for enrichment analyses. List of curated gene signatures defining distinct monocyte subsets used in downstream analyses, including differential expression, module scoring, and Gene Set Enrichment Analysis (GSEA). These signatures were compiled from published datasets. Statistical significance for enrichment analyses using these signatures was assessed by Mann–Whitney U–based scoring with Benjamini–Hochberg FDR correction, as implemented in UCell.

Supplementary Data 2. Gene Ontology (GO) Biological Process enrichment analysis of monocyte-related clusters. Gene Ontology (GO) enrichment analysis identified biological processes significantly associated with monocyte-realted clusters. Enrichment was calculated using Over-Representation Analysis (ORA) performed with clusterProfiler, applying a hypergeometric test with Benjamini–Hochberg false discovery rate (FDR) correction. Only GO terms with adjusted p-value (FDR) < 0.05 are reported.

Supplementary Data 3. GO enrichment analysis for pseudotime Branch 1 and Branch 2. Gene Ontology (GO) Biological Process enrichment was performed on genes associated with pseudotime Branch 1 and Branch 2. Enrichment was calculated using Over-Representation Analysis (ORA) with a hypergeometric test and Benjamini–Hochberg FDR correction.

Supplementary Data 4. IPA biological functions and canonical pathways enriched in Ly6G$^+$ macrophages. Ingenuity Pathway Analysis (IPA) was used to identify enriched biological functions and canonical pathways in Ly6G$^+$ MΦ. Enrichment significance is shown as –$\log_{10}$(Benjamini–Hochberg adjusted p-value), calculated using a hypergeometric enrichment test with Benjamini–Hochberg FDR correction.

Supplementary Data 5. Signatures of human macrophage subsets used for GSEA. List of curated gene signatures defining human macrophage subsets identified across multiple prenatal tissues. These signatures were used for Gene Set Enrichment Analysis (GSEA) to assess enrichment patterns in Ly6G$^+$ macrophages.
