# Supplementary material for: Atypical pericapillary Ly6G⁺Nur77⁺ macrophages initiate type-2 immune responses to allergens in the mouse lung
Source: Nat Commun. 2026 Jan 22;17:1946. doi: 10.1038/s41467-026-68652-5 (PMC12929616; doi:10.1038/s41467-026-68652-5)
Supplement: Supplementary file 8 — Reporting Summary [file 41467_2026_68652_MOESM8_ESM.pdf]

nature portfolio

Corresponding author(s): Beatriz León
Last updated by author(s): Jun 4, 2025



# Reporting Summary

Nature Portfolio wishes to improve the reproducibility of the work that we publish. This form provides structure for consistency and transparency in reporting. For further information on Nature Portfolio policies, see our Editorial Policies and the Editorial Policy Checklist.

## Statistics

For all statistical analyses, confirm that the following items are present in the figure legend, table legend, main text, or Methods section.

| n/a | Confirmed | |
|---|---|---|
| ☐ | ☒ | The exact sample size (*n*) for each experimental group/condition, given as a discrete number and unit of measurement |
| ☐ | ☒ | A statement on whether measurements were taken from distinct samples or whether the same sample was measured repeatedly |
| ☐ | ☒ | The statistical test(s) used AND whether they are one- or two-sided<br>*Only common tests should be described solely by name; describe more complex techniques in the Methods section.* |
| ☐ | ☒ | A description of all covariates tested |
| ☐ | ☒ | A description of any assumptions or corrections, such as tests of normality and adjustment for multiple comparisons |
| ☒ | ☐ | A full description of the statistical parameters including central tendency (e.g. means) or other basic estimates (e.g. regression coefficient) AND variation (e.g. standard deviation) or associated estimates of uncertainty (e.g. confidence intervals) |
| ☒ | ☐ | For null hypothesis testing, the test statistic (e.g. *F*, *t*, *r*) with confidence intervals, effect sizes, degrees of freedom and *P* value noted<br>*Give P values as exact values whenever suitable.* |
| ☒ | ☐ | For Bayesian analysis, information on the choice of priors and Markov chain Monte Carlo settings |
| ☒ | ☐ | For hierarchical and complex designs, identification of the appropriate level for tests and full reporting of outcomes |
| ☒ | ☐ | Estimates of effect sizes (e.g. Cohen's *d*, Pearson's *r*), indicating how they were calculated |

*Our web collection on statistics for biologists contains articles on many of the points above.*

## Software and code

Policy information about availability of computer code

| | |
|---|---|
| Data collection | Attune NxT; BD LSRFortessa; BD FACSAria II; Echo Revolve 4 microscope, SP8 (690/730) confocal microscope (Leica); 10xGenomics Chromium X Controller,;HiSeq 3000 platform, NovaSeq 6000 |
| Data analysis | FlowJo v10.10; Microsoft Excel v16.97.2; Microsoft Word v16.97.2; GraphPad Prism v10.2; fastQC; Trim Galore v0.4.4; STAR v2.5.2a; GSEA v4.3.2; IPA (Qiagen Inc.); Leica Microsystems LAS X v3.5.21594.6;<br>OpenOmics/cell-seek pipeline v2.0.1 and v3.0.2;<br>Cell Ranger v8.0.0 and v9.0.0;<br>R v4.0.2, v4.10.1, v4.3.3, and v4.4.0;<br>Seurat v5.1.0 and v5.2.1;<br>SingleCellExperiment v1.24.0;<br>UCell v2.6.2;<br>Monocle 3 v1.3.7;<br>ComplexHeatmap v2.18.0;<br>MAST;<br>clusterProfiler v4.10.1 and v4.14.6;<br>scCustomize v3.0.1. |

For manuscripts utilizing custom algorithms or software that are central to the research but not yet described in published literature, software must be made available to editors and reviewers. We strongly encourage code deposition in a community repository (e.g. GitHub). See the Nature Portfolio guidelines for submitting code & software for further information.

# Data

Policy information about availability of data

All manuscripts must include a data availability statement. This statement should provide the following information, where applicable:

- Accession codes, unique identifiers, or web links for publicly available datasets
- A description of any restrictions on data availability
- For clinical datasets or third party data, please ensure that the statement adheres to our policy

Data availability
RNA-seq data have been deposited at GEO (GSE291081), and scRNA-seq data have been deposited at GEO (GSE289548) and GEO (GSE305119) and are publicly available as of the date of publication. No new code was included in this study. Any additional information required to reanalyze the data reported in this paper is available from the lead contact upon request.

Code availability
The code used to analyze the scRNA-seq data can be accessed at https://doi.org/10.5281/zenodo.15149503 and https://doi.org/10.5281/zenodo.16848446.

# Research involving human participants, their data, or biological material

Policy information about studies with human participants or human data. See also policy information about sex, gender (identity/presentation), and sexual orientation and race, ethnicity and racism.

| | |
|---|---|
| Reporting on sex and gender | *Use the terms sex (biological attribute) and gender (shaped by social and cultural circumstances) carefully in order to avoid confusing both terms. Indicate if findings apply to only one sex or gender; describe whether sex and gender were considered in study design; whether sex and/or gender was determined based on self-reporting or assigned and methods used. Provide in the source data disaggregated sex and gender data, where this information has been collected, and if consent has been obtained for sharing of individual-level data; provide overall numbers in this Reporting Summary. Please state if this information has not been collected. Report sex- and gender-based analyses where performed, justify reasons for lack of sex- and gender-based analysis.* |
| Reporting on race, ethnicity, or other socially relevant groupings | *Please specify the socially constructed or socially relevant categorization variable(s) used in your manuscript and explain why they were used. Please note that such variables should not be used as proxies for other socially constructed/relevant variables (for example, race or ethnicity should not be used as a proxy for socioeconomic status). Provide clear definitions of the relevant terms used, how they were provided (by the participants/respondents, the researchers, or third parties), and the method(s) used to classify people into the different categories (e.g. self-report, census or administrative data, social media data, etc.) Please provide details about how you controlled for confounding variables in your analyses.* |
| Population characteristics | *Describe the covariate-relevant population characteristics of the human research participants (e.g. age, genotypic information, past and current diagnosis and treatment categories). If you filled out the behavioural & social sciences study design questions and have nothing to add here, write "See above."* |
| Recruitment | *Describe how participants were recruited. Outline any potential self-selection bias or other biases that may be present and how these are likely to impact results.* |
| Ethics oversight | *Identify the organization(s) that approved the study protocol.* |

Note that full information on the approval of the study protocol must also be provided in the manuscript.

# Field-specific reporting

Please select the one below that is the best fit for your research. If you are not sure, read the appropriate sections before making your selection.

☒ Life sciences ☐ Behavioural & social sciences ☐ Ecological, evolutionary & environmental sciences

For a reference copy of the document with all sections, see nature.com/documents/nr-reporting-summary-flat.pdf

# Life sciences study design

All studies must disclose on these points even when the disclosure is negative.

| | |
|---|---|
| Sample size | Sample sizes were determined based on extensive prior experience with similar in vivo allergen exposure and immunological models, ensuring adequate power to detect biologically meaningful differences in outcome measures (such as immune-cell frequencies, cytokine production, or transcriptional responses). For most experiments, 4–8 mice per group were used, which is consistent with established practice in the field and sufficient to detect moderate effect sizes with acceptable variability. No formal statistical power calculation was performed; instead, sample sizes were guided by previously published studies using comparable methodologies and by empirical observations of variability in our own historical datasets. These sample sizes reliably allow detection of reproducible and biologically relevant differences in the context of Type 2 immunity and lung-immune cell responses. |

| | |
|---|---|
| Data exclusions | Data were excluded only in cases of clear technical failure (i.e, unsuccessful tissue harvest, sample loss, or flow cytometry acquisition errors). |
| Replication | All key findings were replicated in at least two independent experiments. For each experiment, multiple biological replicates (individual mice) were used to ensure reproducibility. |
| Randomization | Mice were randomly assigned to groups by genotype and treatment prior to the initiation of the experiment. |
| Blinding | Investigators were not blinded to group allocation during data collection because the experimental procedures (e.g., allergen administration, genetic strain handling, cell sorting) required knowledge of group identity to ensure correct treatment and processing. However, blinding was not relevant to the analytical workflow, as downstream analyses relied on objective, quantitative measurements (e.g., flow cytometry gating, RNA-seq outputs), which were processed using standardized pipelines applied uniformly across all groups. |

# Reporting for specific materials, systems and methods

We require information from authors about some types of materials, experimental systems and methods used in many studies. Here, indicate whether each material, system or method listed is relevant to your study. If you are not sure if a list item applies to your research, read the appropriate section before selecting a response.

## Materials & experimental systems

| n/a | Involved in the study |
|---|---|
| ☐ | ☒ Antibodies |
| ☐ | ☐ Eukaryotic cell lines |
| ☐ | ☐ Palaeontology and archaeology |
| ☐ | ☒ Animals and other organisms |
| ☐ | ☐ Clinical data |
| ☐ | ☐ Dual use research of concern |
| ☐ | ☐ Plants |

## Methods

| n/a | Involved in the study |
|---|---|
| ☐ | ☐ ChIP-seq |
| ☐ | ☒ Flow cytometry |
| ☐ | ☐ MRI-based neuroimaging |

## Antibodies

| | |
|---|---|
| Antibodies used | Anti-B220 (RA3-6B2), BD Biosciences, Cat# 553093, RRID: AB_394622; anti-CD4 (GK1.5), BD Biosciences, Cat# 553729, RRID: AB_395013; anti-CD4 (RM4-5), BD Biosciences, Cat# 550954, RRID: AB_393977 and Cat# 563726, RRID: AB_2738389; anti-CD11a (M17/4), BD Biosciences, Cat# 741071, RRID: AB_2870679; anti-CD11b (M1/70), BD Biosciences, Cat# 553311, RRID: AB_396680 and Cat# 562127, RRID: AB_10896991; anti-CD11c (HL3), BD Biosciences, Cat# 553800, RRID: AB_395059; Cat# 558079, RRID: AB_647251; Cat# 563048, RRID: AB_2734778; anti-CD36 (CRF D-2712), BD Biosciences, Cat# 562702, RRID: AB_2737732; anti-CD43 (S7), BD Biosciences, Cat# 747726, RRID: AB_2872201; anti-CD44 (IM7), BD Biosciences, Cat# 560780, RRID: AB_1937328; anti-CD45.1 (A20), BD Biosciences, Cat# 553775, RRID: AB_10926208 and Cat# 558701, RRID: AB_1645214; anti-CD45.2 (104), BD Biosciences, Cat# 560696, RRID: AB_1727494; Cat# 558702, RRID: AB_1645215; Cat# 563685, RRID: AB_2738374; anti-CD66a (CC1), BD Biosciences, Cat# 750880, RRID: AB_2874976; anti-CD97 (587702), BD Biosciences, Cat# 747935, RRID: AB_2872396; anti-CD103 (M290), BD Biosciences, Cat# 557495, RRID: AB_396732; anti-DCIR4 (MH7E7), BD Biosciences, Cat# 751754, RRID: AB_2875731; anti-Ly6C (AL-21), BD Biosciences, Cat# 560596, RRID: AB_1727555; anti-Ly6G (1A8), BD Biosciences, Cat# 560601, RRID: AB_1727562 and BioLegend, Cat# 127603, RRID: AB_1186105; anti-Siglec-F (E50-2440), BD Biosciences, Cat# 565526, RRID: AB_2739281 and Cat# 565183, RRID: AB_2739097; anti-Siglec-H (440c), BD Biosciences, Cat# 74767, RRID: AB_2744232; anti-Treml4 (16E5), BD Biosciences, Cat# 569880; anti-ALOX5 (ARC1926), Invitrogen, Cat# MA5-38050, RRID: AB_2897968; anti-CD16.2 (FcγRIV), BioLegend, Cat# 149512, RRID: AB_2632745; anti-CD31 (MEC 13.3), BioLegend, Cat# 102516, RRID: AB_2161029; anti-CD49d (R1-2), BioLegend, Cat# 103621, RRID: AB_2565776; anti-CD64 (X54-5/7.1), BioLegend, Cat# 139311, RRID: AB_2563846 and Cat# 139306, RRID: AB_11219391; anti-CD88 (20/70), BioLegend, Cat# 135810, RRID: AB_10900812; anti-CD273 (TY25), BioLegend, Cat# 107218, RRID: AB_2728126; anti-CD274 (10F.9G2), BioLegend, Cat# 124311, RRID: AB_10612935; anti-CX3CR1 (SA011F11), BioLegend, Cat# 149005, RRID: AB_2564314; anti-I-A/I-E (M5/114.15.2), BioLegend, Cat# 107620, RRID: AB_493527; anti-CD115 (AFS98), eBioscience, Cat# 13-1152-85, RRID: AB_466564; anti-CD143 (230214), R&D Systems, Cat# FAB15131R; anti-CD305 (113), eBioscience, Cat# 12-3051-82, RRID: AB_1210738; anti-CCR7 (4B12), eBioscience, Cat# 13-1971-82, RRID: AB_466642; anti-F4/80 (BM8), eBioscience, Cat# 11-4801-82, RRID: AB_2637191 and Cat# 12-4801-82, RRID: AB_465923; anti-MertK (DS5MMER), eBioscience, Cat# 17-5751-82, RRID: AB_2716943; anti-IL-13 (eBio13A), eBioscience, Cat# 12-7133-82, RRID: AB_763559; anti-IL-5 (TRFK5), BioLegend, Cat# 504311, RRID: AB_2563161; anti-IL-17 (TC11-18H10.1), BioLegend, Cat# 506941, RRID: AB_2565836; anti-Ki-67 (16A8), BioLegend, Cat# 652406, RRID: AB_2561930; anti-IFNγ (XMG1.2), BD Biosciences, Cat# 557649, RRID: AB_396766; anti-Streptavidin, Fisher Scientific, Cat# 565144, RRID: AB_2869657; Cat# 560797, RRID: AB_2033992; Cat# S-32355; anti-CCR2 (475301), R&D Systems, Cat# FAB5538A100; anti-GFP, Life Technologies, Cat# A21311, RRID: AB_221477; anti-PAR2 (SAM11), Santa Cruz Biotechnology, Cat# sc-13504; DAPI Solution, BD Fisher, Cat# BDB564907. All flow cytometry antibodies were used at manufacturer-recommended concentrations (typically 1:100–1:200 for surface staining and 1:50–1:100 for intracellular staining). |
| Validation | All primary antibodies used in this study have been validated by the manufacturers for flow cytometry and/or immunofluorescence in mouse tissues. Antibody clones were selected based on extensive prior use in the literature and established specificity for their respective targets. |

# Eukaryotic cell lines

Policy information about cell lines and Sex and Gender in Research

| | |
|---|---|
| Cell line source(s) | *State the source of each cell line used and the sex of all primary cell lines and cells derived from human participants or vertebrate models.* |
| Authentication | *Describe the authentication procedures for each cell line used OR declare that none of the cell lines used were authenticated.* |
| Mycoplasma contamination | *Confirm that all cell lines tested negative for mycoplasma contamination OR describe the results of the testing for mycoplasma contamination OR declare that the cell lines were not tested for mycoplasma contamination.* |
| Commonly misidentified lines (See ICLAC register) | *Name any commonly misidentified cell lines used in the study and provide a rationale for their use.* |

# Palaeontology and Archaeology

| | |
|---|---|
| Specimen provenance | *Provide provenance information for specimens and describe permits that were obtained for the work (including the name of the issuing authority, the date of issue, and any identifying information). Permits should encompass collection and, where applicable, export.* |
| Specimen deposition | *Indicate where the specimens have been deposited to permit free access by other researchers.* |
| Dating methods | *If new dates are provided, describe how they were obtained (e.g. collection, storage, sample pretreatment and measurement), where they were obtained (i.e. lab name), the calibration program and the protocol for quality assurance OR state that no new dates are provided.* |

☐ Tick this box to confirm that the raw and calibrated dates are available in the paper or in Supplementary Information.

| | |
|---|---|
| Ethics oversight | *Identify the organization(s) that approved or provided guidance on the study protocol, OR state that no ethical approval or guidance was required and explain why not.* |

Note that full information on the approval of the study protocol must also be provided in the manuscript.

# Animals and other research organisms

Policy information about studies involving animals; ARRIVE guidelines recommended for reporting animal research, and Sex and Gender in Research

| | |
|---|---|
| Laboratory animals | All mice were Mus musculus, obtained from The Jackson Laboratory (JAX) unless otherwise indicated. The exact strain name, genetic background, and JAX stock number are listed below: C57BL/6J (B6) — JAX Stock #000664; inbred strain., B6.SJL-Ptprca Pepcb/BoyJ (CD45.1+ B6 congenic) — JAX Stock #002014; congenic on C57BL/6 background, C57BL/6-Tg(TcraTcrb)425Cbn/J (OTII) — JAX Stock #004194; transgenic OVA-specific CD4 TCR, B6.129-Il4tm1Lky/J (B6.4get IL-4-GFP reporter) — JAX Stock #004190; reporter on C57BL/6 background, B6.Cg-F2rl1tm1Mslb/J (Par2-/-)— JAX Stock #004993; targeted deletion on C57BL/6 background, C57BL/6-Rr39em1Ched/J (Nr4a1se_2/se_2) — JAX Stock # 030204; SE-2 Nur77 mutant on C57BL/6 background, B6;129S2-Nr4a1tm1Jmi/J (Nur77-/-)— JAX Stock #006187; knockout on on C57BL/6 background, B6.129S4-Ccr2tm1Ifc/J (Ccr2-/-)>) — JAX Stock #004999; knockout on on C57BL/6 background, C57BL/6-Tg(Nr4a1-EGFP/cre)820Khog/J (Nur77GFP) — JAX Stock #016617; reporter/Cre strain on C57BL/6 background, B6(Cg)-Tlr4tm1.2Karp/J (Tlr4-/-)— JAX Stock #029015; targeted deletion on C57BL/6 background, B6;129S2-Alox5tm1Fun/J (Alox5-/-)— JAX Stock #004155; knockout on C57BL/6 background, C57BL/6J-Ms4a3em2(cre)Fgnx/J (Ms4a3cre) — JAX Stock #036382; CRISPR-engineered Cre driver on C57BL/6 background, and B6.Cg-Gt(ROSA)26Sortm9(CAG-tdTomato)Hze/J (Ai9) — JAX Stock #007909; Cre-dependent tdTomato reporter on C57BL/6 background. |
| Wild animals | No wild animals were used in this study |
| Reporting on sex | Both male and female mice were used in this study. However, the study was not specifically powered or designed to detect sex-based differences. As such, findings were not stratified by sex and conclusions apply to both sexes collectively. |
| Field-collected samples | No field collected samples in this study |
| Ethics oversight | Animals were housed under specific pathogen-free conditions, and all experiments conformed to ethical guidelines and were approved by the Institutional Animal Care and Use Committee (IACUC) of the National Institute of Allergy and Infectious Diseases (NIAID), NIH, under protocols assigned to the Laboratory of Allergic Diseases (LAD), and by the Institutional Animal Care and Use Committee of the University of Alabama at Birmingham (UAB). |

Note that full information on the approval of the study protocol must also be provided in the manuscript.

# Clinical data

Policy information about clinical studies

All manuscripts should comply with the ICMJE guidelines for publication of clinical research and a completed CONSORT checklist must be included with all submissions.

Clinical trial registration — *Provide the trial registration number from ClinicalTrials.gov or an equivalent agency.*

Study protocol — *Note where the full trial protocol can be accessed OR if not available, explain why.*

Data collection — *Describe the settings and locales of data collection, noting the time periods of recruitment and data collection.*

Outcomes — *Describe how you pre-defined primary and secondary outcome measures and how you assessed these measures.*

# Dual use research of concern

Policy information about dual use research of concern

## Hazards

Could the accidental, deliberate or reckless misuse of agents or technologies generated in the work, or the application of information presented in the manuscript, pose a threat to:

| No | Yes | |
|---|---|---|
| ☐ | ☐ | Public health |
| ☐ | ☐ | National security |
| ☐ | ☐ | Crops and/or livestock |
| ☐ | ☐ | Ecosystems |
| ☐ | ☐ | Any other significant area |

## Experiments of concern

Does the work involve any of these experiments of concern:

| No | Yes | |
|---|---|---|
| ☐ | ☐ | Demonstrate how to render a vaccine ineffective |
| ☐ | ☐ | Confer resistance to therapeutically useful antibiotics or antiviral agents |
| ☐ | ☐ | Enhance the virulence of a pathogen or render a nonpathogen virulent |
| ☐ | ☐ | Increase transmissibility of a pathogen |
| ☐ | ☐ | Alter the host range of a pathogen |
| ☐ | ☐ | Enable evasion of diagnostic/detection modalities |
| ☐ | ☐ | Enable the weaponization of a biological agent or toxin |
| ☐ | ☐ | Any other potentially harmful combination of experiments and agents |

# Plants

Seed stocks — *Report on the source of all seed stocks or other plant material used. If applicable, state the seed stock centre and catalogue number. If plant specimens were collected from the field, describe the collection location, date and sampling procedures.*

Novel plant genotypes — *Describe the methods by which all novel plant genotypes were produced. This includes those generated by transgenic approaches, gene editing, chemical/radiation-based mutagenesis and hybridization. For transgenic lines, describe the transformation method, the number of independent lines analyzed and the generation upon which experiments were performed. For gene-edited lines, describe the editor used, the endogenous sequence targeted for editing, the targeting guide RNA sequence (if applicable) and how the editor was applied.*

Authentication — *Describe any authentication procedures for each seed stock used or novel genotype generated. Describe any experiments used to assess the effect of a mutation and, where applicable, how potential secondary effects (e.g. second site T-DNA insertions, mosiacism, off-target gene editing) were examined.*

# ChIP-seq

## Data deposition

☐ Confirm that both raw and final processed data have been deposited in a public database such as GEO.

☐ Confirm that you have deposited or provided access to graph files (e.g. BED files) for the called peaks.

| | |
|---|---|
| Data access links<br>*May remain private before publication.* | *For "Initial submission" or "Revised version" documents, provide reviewer access links. For your "Final submission" document, provide a link to the deposited data.* |
| Files in database submission | *Provide a list of all files available in the database submission.* |
| Genome browser session<br>(e.g. UCSC) | *Provide a link to an anonymized genome browser session for "Initial submission" and "Revised version" documents only, to enable peer review. Write "no longer applicable" for "Final submission" documents.* |

## Methodology

| | |
|---|---|
| Replicates | *Describe the experimental replicates, specifying number, type and replicate agreement.* |
| Sequencing depth | *Describe the sequencing depth for each experiment, providing the total number of reads, uniquely mapped reads, length of reads and whether they were paired- or single-end.* |
| Antibodies | *Describe the antibodies used for the ChIP-seq experiments; as applicable, provide supplier name, catalog number, clone name, and lot number.* |
| Peak calling parameters | *Specify the command line program and parameters used for read mapping and peak calling, including the ChIP, control and index files used.* |
| Data quality | *Describe the methods used to ensure data quality in full detail, including how many peaks are at FDR 5% and above 5-fold enrichment.* |
| Software | *Describe the software used to collect and analyze the ChIP-seq data. For custom code that has been deposited into a community repository, provide accession details.* |

# Flow Cytometry

## Plots

Confirm that:

☐ The axis labels state the marker and fluorochrome used (e.g. CD4-FITC).

☒ The axis scales are clearly visible. Include numbers along axes only for bottom left plot of group (a 'group' is an analysis of identical markers).

☒ All plots are contour plots with outliers or pseudocolor plots.

☒ A numerical value for number of cells or percentage (with statistics) is provided.

## Methodology

| | |
|---|---|
| Sample preparation | Lungs were isolated, cut into small fragments, and digested for 45 min at 37°C with 0.6 mg/mL collagenase A (Sigma) and 30 mg/mL DNase I (Sigma) in RPMI-1640 medium (GIBCO). Digested lungs or mLNs were mechanically disrupted by passage through a wire mesh. Blood was collected in Dextran-EDTA buffer. Bone marrow was collected from the femurs and tibias and filtered through a wire mesh. Red blood cells were lysed with 150 mM $NH_4Cl$, 10 mM $KHCO_3$, and 0.1 mM EDTA. |
| Instrument | Data were acquired using an Attune NxT or BD LSRFortessa instrument. |
| Software | Flow cytometry data were analyzed using FlowJo (v10.10). |
| Cell population abundance | A BD FACSAria II was used to sort allergen$^{+}$ cells and defined populations from lung and mLNs. Post-sort purity was >98%. |
| Gating strategy | A detailed gating strategy for FACS analysis is provided in all relevant experiemnts |

☒ Tick this box to confirm that a figure exemplifying the gating strategy is provided in the Supplementary Information.

# Magnetic resonance imaging

## Experimental design

| | |
|---|---|
| Design type | *Indicate task or resting state; event-related or block design.* |
| Design specifications | *Specify the number of blocks, trials or experimental units per session and/or subject, and specify the length of each trial or block (if trials are blocked) and interval between trials.* |

Behavioral performance measures | *State number and/or type of variables recorded (e.g. correct button press, response time) and what statistics were used to establish that the subjects were performing the task as expected (e.g. mean, range, and/or standard deviation across subjects).*

## Acquisition

Imaging type(s) | *Specify: functional, structural, diffusion, perfusion.*

Field strength | *Specify in Tesla*

Sequence & imaging parameters | *Specify the pulse sequence type (gradient echo, spin echo, etc.), imaging type (EPI, spiral, etc.), field of view, matrix size, slice thickness, orientation and TE/TR/flip angle.*

Area of acquisition | *State whether a whole brain scan was used OR define the area of acquisition, describing how the region was determined.*

Diffusion MRI ☐ Used ☐ Not used

## Preprocessing

Preprocessing software | *Provide detail on software version and revision number and on specific parameters (model/functions, brain extraction, segmentation, smoothing kernel size, etc.).*

Normalization | *If data were normalized/standardized, describe the approach(es): specify linear or non-linear and define image types used for transformation OR indicate that data were not normalized and explain rationale for lack of normalization.*

Normalization template | *Describe the template used for normalization/transformation, specifying subject space or group standardized space (e.g. original Talairach, MNI305, ICBM152) OR indicate that the data were not normalized.*

Noise and artifact removal | *Describe your procedure(s) for artifact and structured noise removal, specifying motion parameters, tissue signals and physiological signals (heart rate, respiration).*

Volume censoring | *Define your software and/or method and criteria for volume censoring, and state the extent of such censoring.*

## Statistical modeling & inference

Model type and settings | *Specify type (mass univariate, multivariate, RSA, predictive, etc.) and describe essential details of the model at the first and second levels (e.g. fixed, random or mixed effects; drift or auto-correlation).*

Effect(s) tested | *Define precise effect in terms of the task or stimulus conditions instead of psychological concepts and indicate whether ANOVA or factorial designs were used.*

Specify type of analysis: ☐ Whole brain ☐ ROI-based ☐ Both

Statistic type for inference | *Specify voxel-wise or cluster-wise and report all relevant parameters for cluster-wise methods.*

(See Eklund et al. 2016)

Correction | *Describe the type of correction and how it is obtained for multiple comparisons (e.g. FWE, FDR, permutation or Monte Carlo).*

## Models & analysis

| n/a | Involved in the study |
| --- | --- |
| ☐ | ☐ Functional and/or effective connectivity |
| ☐ | ☐ Graph analysis |
| ☐ | ☐ Multivariate modeling or predictive analysis |

Functional and/or effective connectivity | *Report the measures of dependence used and the model details (e.g. Pearson correlation, partial correlation, mutual information).*

Graph analysis | *Report the dependent variable and connectivity measure, specifying weighted graph or binarized graph, subject- or group-level, and the global and/or node summaries used (e.g. clustering coefficient, efficiency, etc.).*

Multivariate modeling and predictive analysis | *Specify independent variables, features extraction and dimension reduction, model, training and evaluation metrics.*
